# The AmP project: Comparing species on the basis of dynamic energy budget parameters

**DOI:** 10.1371/journal.pcbi.1006100

**Published:** 2018-05-09

**Authors:** Gonçalo M. Marques, Starrlight Augustine, Konstadia Lika, Laure Pecquerie, Tiago Domingos, Sebastiaan A. L. M. Kooijman

**Affiliations:** 1 MARETEC – Marine, Environment & Technology Center, Instituto Superior Técnico, Universidade de Lisboa, Lisboa, Portugal; 2 Akvaplan-niva, Fram High North Research Centre for Climate and the Environment, Tromsø, Norway; 3 Department of Biology, University of Crete, Heraklion, Greece; 4 LEMAR, IRD, CNRS, UBO, Ifremer, Plouzané, France; 5 Department of Theoretical Biology, VU University Amsterdam, Amsterdam, The Netherlands; University of Michigan, UNITED STATES

## Abstract

We developed new methods for parameter estimation-in-context and, with the help of 125 authors, built the AmP (Add-my-Pet) database of Dynamic Energy Budget (DEB) models, parameters and referenced underlying data for animals, where each species constitutes one database entry. The combination of DEB parameters covers all aspects of energetics throughout the full organism’s life cycle, from the start of embryo development to death by aging. The species-specific parameter values capture biodiversity and can now, for the first time, be compared between animals species. An important insight brought by the AmP project is the classification of animal energetics according to a family of related DEB models that is structured on the basis of the mode of metabolic acceleration, which links up with the development of larval stages. We discuss the evolution of metabolism in this context, among animals in general, and ray-finned fish, mollusks and crustaceans in particular. New DEBtool code for estimating DEB parameters from data has been written. AmPtool code for analyzing patterns in parameter values has also been created. A new web-interface supports multiple ways to visualize data, parameters, and implied properties from the entire collection as well as on an entry by entry basis. The DEB models proved to fit data well, the median relative error is only 0.07, for the 1035 animal species at 2018/03/12, including some extinct ones, from all large phyla and all chordate orders, spanning a range of body masses of 16 orders of magnitude. This study is a first step to include evolutionary aspects into parameter estimation, allowing to infer properties of species for which very little is known.

## Author summary

We discovered that parameters of Dynamic Energy Budget (DEB) models can be estimated from a set of simple data on animal life history aspects, growth and reproduction, if treated in combination. Apart from goodness-of-fit as an estimation criterion, relations with parameter values of other species are important, since DEB parameters have a clear physiological interpretation and a good fit for the wrong reasons is always a risk to consider. We developed and optimized methods for this type of parameter estimation-in-context and organized the results of over 1000 animal species in the open-access Add-my-Pet (AmP) database, to which 125 authors contributed so far. We also developed software package AmPtool to compare parameter values in the collection, that builds on DEBtool to assist applications of DEB theory. A family of related DEB models, structured with respect to the modes of metabolic acceleration, captures biodiversity, including various life stages. We discuss some features of the family structure of DEB models in an evolutionary context. The AmP collection has a great potential for research on the role of biodiversity in ecosystem structure and functioning, which will grow with the size of the database.

## Introduction

The role of biodiversity in ecosystem structure and functioning is central for conservation and environmental quality management, as well as biospherics and earth system studies. Biodiversity is not only about the number of species present, but also the number and nature of the different characteristics and functions which make up a community or an ecosystem, often referred to as traits. Scientists and managers are turning towards such trait-based approaches to measure the health and vitality of ecosystems. In this context of apprehending biodiversity on the basis of diversity of characteristics and functionalities we have been developing the AmP (Add-my-Pet) project.

AmP is a database of referenced data on animal energetics, parameter values of models based on Dynamic Energy Budget (DEB) theory [1–4], and properties derived from these parameters. Some 125 authors contributed to the database at 2018/03/12. The AmP project aims: (i) to find the simplest organization principles for metabolism upon which all life is based and (ii) to understand taxon-specific patterns as variations on this common organization. The development of DEB theory started in 1979 and meanwhile over 700 papers have been published on DEB theory, see www.zotero.org/groups/500643/deb_library/.

Partly based on the fact that a large number of popular empirical models turned out to be special cases of DEB models [4], we claim that DEB theory is presently the best tested quantitative theory in biology. The comparison of species on the basis of parameter values is an important aspect of the AmP project. Species-comparisons based on measured quantities suffer from the problem that these quantities typically have contributions from many underlying interacting processes, and were not measured for all species of interest. Parameters of mechanistic models, however, have much simpler links with such processes, which makes it easier to find explanations for differences between species. Moreover, the complete parameter set is available for each entry, allowing to predict, e.g. respiration, without any measured data on respiration.

Comparison of species is, however, not the only important application of the AmP website. Prediction of effects of global change [5], understanding the geographic distribution of species [6–8], the effects of (toxic) chemical compounds [9–12], the optimization of bio-production (e.g. aquaculture and agriculture [13–15]), stock management, the best re-introduction of endangered species or the control of invading species [16], are just examples of applications where detailed knowledge of energetics of species in a DEB context is very useful. Like many ecologists, we see energetics as the key to understand the ecological behavior of species [17], and as the root of population [18] and ecosystem dynamics [19, 20], with consequences at the planetary level [21]. This is the context that motivated the development of DEB theory, of which the AmP project is an application. In view of the rapid build-up of ecological stress all over the world, we think that the field is in urgent need of an online database like AmP.

AmP started in 2009 as an educational initiative, to teach researchers how to estimate DEB parameters from their data and animals have the simplest metabolisms (if we compare with say plants, bacteria or microalgae). The database grew and in 2013, (at about 300 entries) we teamed up, formed the first AmP curator board, and together developed the code and web-platform underlying AmP. We wanted to get an overview on: (i) how well the standard DEB model worked for describing animal metabolism, (ii) standardize and improve the parameter estimation procedure and (iii) improve our capacity to judge the realism of parameter values.

We start with a brief introduction of DEB theory. All applications of models, including testing of the model against data, start with knowledge of parameter values. The parameters of a DEB model must be estimated from a collection of data sets on the various aspects of energy budgets and life history, using all this information in combination. This involves new features in the methodology of parameter estimation. The next section of this paper describes improvements we implemented, based on the co-variation method [22, 23], which was used in an early phase of the database project.

Moreover we needed more administrative rigor and improved methods for detecting patterns in parameter values. Development of new routines and re-organization of the previous estimation procedure allowed us to include these new extensions. So the following section describes the new web-interface and structure of the database. Finally we present and discuss the results obtained after implementing the new method and reaching 1035 species in the collection at 2018/03/12. Given that these entries employ together 270 different types of data, in 585 combinations, the estimation of 14 parameters of each species not only illustrates the scope of the data reduction, but also the step-up in comparison potential.

## Methods

### DEB theory

**Fig 1:**
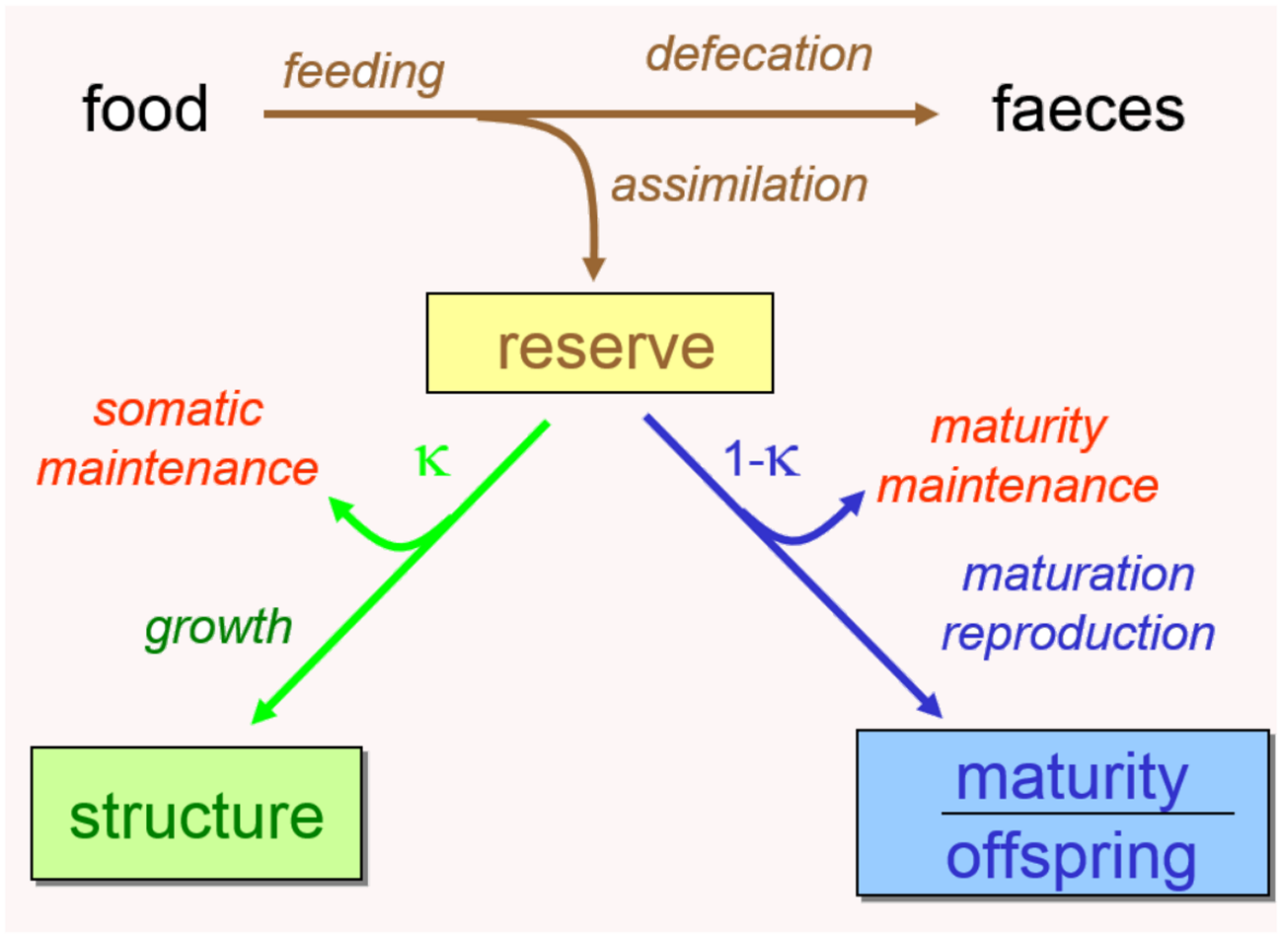
Model scheme of the standard DEB model. Boxes: state variables. Arrows: mass/ energy fluxes associated with each of the processes specified on the scheme. Each process is quantified by a model parameter (see Table 1). The only process that is not represented here is aging, which is quantified by two parameters.

Dynamic Energy Budget (DEB) theory, aims to specify commonalities underlying metabolic organization for all life. It does this by delimiting a small set of assumptions from which mathematical formulae for metabolism are derived, covering the start of embryo development to death by aging through a range of life stages [24–27]. DEB models are meant to apply to all life on earth and allow species comparisons on the basis of (functions of) parameters of that model. The standard DEB model (’std’) is the simplest non-degenerated DEB model implied by the theory and it applies to heterotrophic animals (see Fig 1 for the summary). We refer the reader to [28] for an accessible summary of the principles of DEB theory and a description of the standard DEB model. A detailed derivation of the model on the basis of underlying assumptions is presented in [4], chap. 2. Each DEB parameter of the std-DEB model (see Table 1) has a clear link with one underlying physiological process (specified on the arrows of Fig 1). The combination of parameters covers all aspects of energetics throughout the full life cycle of organisms, include feeding, digestion, storage, maintenance, growth, development, reproduction, aging. Parameter values are individual-specific in the context of DEB theory, but the difference between individuals are typically small enough to average for a species in a meaningful way. Parameter values determine how state variables of an individual (reserve, structure, maturity and reproduction buffer) change in time through all life stages (embryo, juvenile, adult). Life stages have specific definitions: Embryos do not assimilate; Juveniles assimilate and allocate to maturity but not to reproduction; Adults feed, no longer allocate to maturity but store energy/ mass allocated to reproduction into a reproduction buffer which is converted to offspring. To complete the background information needed for this study, we must point to the co-variation rules [29]. If there was no selection pressure of parameter values then particular parameters would co-vary in a simple way with the dimensionless zoom factor *z* (Table 1), purely based on parameters being either intensive or extensive. The estimation of parameters for each species allows comparing parameters between species and seeing to what extent each parameter is in fact either dependent or independent of body size for different taxonomic groups, thus revealing important patterns in how environmental pressures exert selection pressure on parameter values.

**Table 1:** The 14 primary parameters of the std-DEB model in a time-length-energy frame [4]. The values are considered typical values among species at 20°C with maximum structural length $L_{m}=zL_{m}^{\text{ref}}$ for a dimensionless zoom factor *z* and $L_{m}^{\text{ref}}=1$ cm. Structural length is the cubic root of structural volume, which, together with reserve, contributes to body mass. The Gompertz stress coefficient is almost zero for ectotherms and around 0.1 for endotherms, while the Weibull aging acceleration varies greatly between species.

specific searching rate	$\left\{{\dot{F}}_{m}\right\}$	6.5 l cm^−2^ d^−1^
assimilation efficiency	*κ*_*X*_	0.8
max surface-spec. assimilation rate	$\left\{{\dot{p}}_{Am}\right\}$	22.5 *z* J cm^−2^d^−1^
energy conductance	$\dot{v}$	0.02 cm d^−1^
allocation fraction to soma	*κ*	0.8
reproduction efficiency	*κ*_*R*_	0.95
volume-spec. som. maint. cost	$\left[{\dot{p}}_{M}\right]$	18 J cm^−3^d^−1^
surface-spec. som. maint. cost	$\left\{{\dot{p}}_{T}\right\}$	0 J cm^−2^d^−1^
maturity maint. rate coeff.	${\dot{k}}_{J}$	0.002 d^−1^
specific cost for structure	[*E*_*G*_]	2800 J cm^−3^
maturity at birth	$E_{H}^{b}$	0.275 *z*^3^ J
maturity at puberty	$E_{H}^{p}$	166 *z*^3^ J
Gombertz stress coefficient	*s*_*G*_	0.0001
Weibull aging acceleration	${\ddot{h}}_{a}$	10^−10^ d^−2^

### An improved parameter estimation method

Prior to this work, DEB parameters for the AmP entries were obtained with the covariation-method for parameter estimation [22, 23]. All the code for parameter estimation is developed in the DEBtool_M package, which is frequently updated and freely available at https://github.com/add-my-pet/DEBtool_M/. In this section we describe improvements to the method which are implemented in DEBtool. The improvements comprise:

- organization of data, parameters and model predictions into three user-defined functions which are controlled by a single ‘run-file’ where all estimation options are set and the estimation is performed.
- Automatized saving of parameter estimates and possibility to use the saved values as a new seed parameter set.
- A new and much better loss function that is minimized to estimate parameters which is symmetric in the role of data and predictions, and which addresses differences in dimensions.
- Automatized setting of weight coefficients, chemical parameters, and pseudo-data.
- Application of filters in a simplex method to confine parameter trajectories to the physically allowed part of the parameter space.

**Fig 2:**
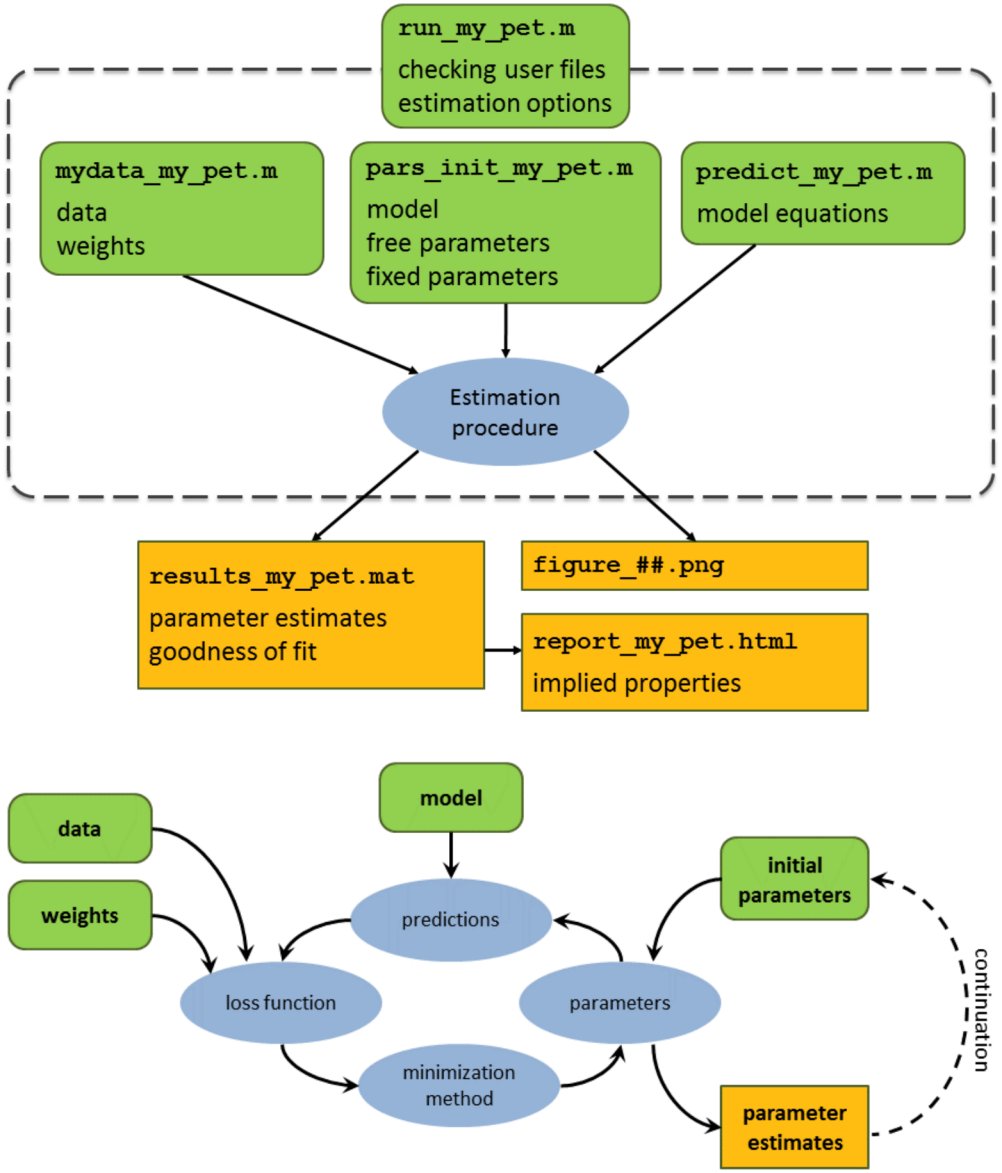
Top figure: Architecture of the AmP procedure. The rounded (green) rectangles represent files that are set by the user: run_my_pet, mydata_my_pet, pars_init_my_pet, predict_my_pet. The specific inputs for each file are specified (see text for details). For example: the user must set data and weights in mydata_my_pet etc. The (orange) rectangles represent the output. All the estimated parameters are stored in results_my_pet.mat. Figures can be stored as .png. Parameters in the .mat file can be used to generate an html file which reports all types of DEB quantities. The estimation procedure (blue oval) is further detailed in the bottom figure. Bottom: conceptual overview of the four elements underlying the AmP parameter estimation procedure (blue ovals). The information that is used as input for each of the elements is represented in rounded (green) rectangles. The elements represented by the ovals perform the computations. The output of the estimation procedure (the parameters) is represented by the (orange) rectangle. The results returned by the procedure can then be used to restart the procedure (continuation method described in the text).

The overview of the parameter estimation procedure is presented in Fig 2. In the next section we describe the important new components.

#### User defined files

Parameters for a species are estimated using four user defined files: mydata_my_pet, pars_init_my_pet, predict_my_pet, and run_my_pet (green rounded boxes in Fig 2, top panel). The ‘my_pet’ in each of the four user-defined file names must be replaced with the Genus and species name that is being estimated. For example, mydata_my_pet is renamed mydata_Daphnia_magna when one estimates parameters for that species. The mydata-, pars_init- and predict- files are user defined functions containing user-defined information specified in Fig 2, top panel (and further described in the next subsections). The parameter estimation procedure (blue oval Fig 2, top panel) is controlled via the run-file. The run-file is where the user sets all of the estimation options (which will also be detailed below). The user also decides in the run-file whether to save or not the parameters (and figures) in a ‘results_my_pet.mat’ file (and .png images).

#### Data

All of the data are set in the user defined mydata-file (Fig 2). The AmP procedure distinguishes between data (referenced eco-physiological measurements), meta-data, auxiliary-data and pseudo-data. Data are further divided into zero- and uni-variate data, see section 3.1 [22]. The improved estimation routine can handle the situation where zero-variate data take value 0. There is a strict standardization of labels for each type of real-data (see tables 1 and 2 in [30]). Once all of the data is entered in the mydata-file, the completeness of the data is assessed (on a scale of 1 to 10) based on how many different aspects of metabolic performance are available. The way to score data completeness is provided in [22, table 3]. Zero-variate data contain both real- as well as pseudo-data. The concept of pseudo-data is introduced in [22, section 2.3] and the role of pseudo-data is studied extensively in [23, section 3.2]. In brief, pseudo-data constitute a set of fixed (functions of) parameter values for a generalized animal species (Table 2) that is treated as data (from which predictions can deviate) and do not depend on maximum body size. Actual animal species are thought to deviate from the generalized one mainly by their maximum body size, causing deviations in particular parameters, as specified by the co-variation rules. Pseudo-data serve as prior knowledge on parameter values when the data do not contain that information. The impact of (pseudo)data on the resulting parameter estimates is controlled by weight coefficients. The default weight-coefficients for pseudo-data are set an order of magnitude smaller than that for data to make sure that if data determine parameters well, pseudo-data hardly contribute. Meta-data comprise information relating to the species classification, author names and affiliations, submission and acceptance dates, lists of labels which specify the different type of real data that were used, and references for the real-data which were used. Auxiliary-data contain additional information on the real-data that are needed for composing predictions for the data, such as temperature or food level (and how they change in time).

**Table 2:** Default weight settings for the pseudo-data according to loss functions “sb” or “su”, see Methods (loss function) for their definition.

Parameter	Value	Unit	sb	su
$\dot{v}$	0.02	cm/d	0.1	0.00001
*κ*	0.8	-	0.1	0.1
*κ*_*R*_	0.95	-	0.1	0.1
${\dot{p}}_{M}$	18	J/d/cm^3^	0.1	0.00001
${\dot{k}}_{J}$	0.002	1/d	0.1	0.00001
*κ*_*G*_	0.8	-	20	20

#### Initial parameter set

Parameter values are set in the pars_init-file (see Fig 2). They can either be free or fixed. Free means that they are estimated, fixed means that they are not. The choice to free or fix a parameter depends on data availability. Primary parameters of the std-DEB model are listed in Table 1. The new system includes chemical parameters to the DEB parameters list. These chemical parameters include the relative elemental frequency of food, reserve, structure and feces (see Fig 1), chemical potentials and water content. In this new system, the default setting for water content depends on the phylum, sometimes also on the class. The default values for the chemical parameters and pseudo-data are realistic for most species, but can be overwritten. The chemical parameters are typically kept fixed, although the procedure allows their estimation, if sufficient information is included in the data. The (non-chemical) initial parameter set is either specified manually, inspired by related species that are present in the collection, or else computed automatically using the zero variate data for the species. The automatic computation is based on the bijection of 9 data points to 9 parameters, see [31].

#### Loss function

Several loss functions can now be selected for the estimation procedure:

- “sb”, symmetric bounded (by product): $\sum_{i=1}^{n}\sum_{j=1}^{n_{i}}w_{ij}\frac{{\left(d_{ij}-p_{ij}\right)}^{2}}{d_{i}^{2}+p_{i}^{2}}$ described in [32]
- “su”, symmetric unbounded (by product): $\sum_{i=1}^{n}\sum_{j=1}^{n_{i}}w_{ij}{\left(d_{ij}-p_{ij}\right)}^{2}(\frac{1}{d_{i}^{2}}+\frac{1}{p_{i}^{2}})$ described in [32]
- “re”, relative error (symmetric by addition): $\sum_{i=1}^{n}\sum_{j=1}^{n_{i}}w_{ij}\frac{{\left(d_{ij}-p_{ij}\right)}^{2}}{d_{i}^{2}}$ described in [22, section 3.2]

where *i* refers to the data set and *j* to a given point in data set *i*. *d*_*ij*_ stands for the data, *p*_*ij*_ for the model prediction and *w*_*ij*_ for the associated weight coefficient. Finally, *d*_*i*_ and *p*_*i*_ in the denominator represent, respectively, the average of all data points (*d*_*ij*_) and predictions (*p*_*ij*_) in set *i*: $d_{i}=\frac{1}{n_{i}}\sum_{j=1}^{n_{i}}d_{ij}$ and $p_{i}=\frac{1}{n_{i}}\sum_{j=1}^{n_{i}}p_{ij}$. *w*_*ij*_ are weight coefficients which are defined in the next section. The different loss functions differ in how data and predictions contribute. The location of the minimum of the loss function depends on both the choices of loss function and the values of the weight coefficients. The more technical aspects of estimating parameters are discussed in [32].

#### Weight coefficients

The weight coefficients serve to (subjectively) quantify the confidence of the user in the data-sets as well as for specific data points. The weight coefficients are automatically set to *w*_*ij*_ = 1/*n*_*i*_ where *i* designates the data set and *j* the point on data set *i*, and *n*_*i*_ designates the number of points in data set *i*. The motivation is to ensure that each data set contributes equally to the loss function (instead of each data point contributing equally). The user can overwrite default weight values (for either the whole data set or else for particular values). This is done in the mydata-file (see Fig 2). The overwriting of the weight coefficient is done by multiplying the default value by a dimensionless factor. This allows including user-based knowledge about accuracy of data. The AmP procedure distinguishes between how real and pseudo data are weighted such that if data determine parameters well, pseudo-data hardly contribute. The AmP estimation procedure includes several loss functions. The user defines which loss function to use in estimation options—the default weight coefficients for pseudo-data depends on which loss function is being used (these defaults are presented in the Results).

#### Filters

The improved parameter estimation contains filters. Parameter filters are coded constraints that test parameter values for consistency during the estimation process, e.g. birth and puberty must be reachable, most parameters cannot become negative, and fractions are bounded between 0 and 1. The check is now automatically performed in the downhill simplex method (Nelder-Mead) by calling the filter before any call to the predict-function (see Fig 2). In the case the new simplex point does not pass it, it is discarded and another is selected, without calling the predict-function. The user can insert additional ‘customized’ filters in the predict-function. The algorithm underlying the constraints in parameter space (as well as underlying assumptions) are detailed in [31]. In the results section, we report that the standard DEB model needs particular extensions according to taxonomic group. Each model extension is provided with a unique label and filters were developed for each label. These are presented in the results and discussed in the final section of this study.

### Goodness of fit criterion

One way to judge how good the parameter estimates are is to compute a goodness of fit measure which assesses how close the model predictions are to all of the data. Goodness of fit is not enough, one also needs to check for biological realism. The previous system used goodness of fit measures defined in section 2 of [23]. The new system uses the Mean Relative Error (MRE) and the Symmetric Mean Squared Error (SMSE) to quantify the goodness of fit. $\text{MRE}=\frac{1}{n^{\prime }}\;\sum_{i=1}^{n}{\text{RE}}_{i}$, where ${\text{RE}}_{i}=\sum_{j=1}^{n_{i}}\;\frac{w_{ij}}{w_{i}}\frac{|p_{ij}-d_{ij}|}{|d_{i}|}$ ($w_{i}=\sum_{j=1}^{n_{i}}\;w_{ij}>0$), that simplifies to $RE_{i}=\left|\frac{p_{i1}}{d_{i1}}-1\right|$ for zero-variate data. *n*′ is the number of data sets with *w*_*i*_ greater than 0. $\text{SMSE}=\frac{1}{n^{\prime }}\sum_{i=1}^{n}{\text{SSE}}_{i}$ where ${\text{SSE}}_{i}=\sum_{j=1}^{n_{i}}\frac{w_{ij}}{w_{i}}\frac{{\left(p_{ij}-d_{ij}\right)}^{2}}{d_{i}^{2}+p_{i}^{2}}$, that simplifies to ${\text{SSE}}_{i}=\frac{{\left(p_{i1}/d_{i1}-1\right)}^{2}}{1+{\left(p_{i1}/d_{i1}\right)}^{2}}$ for zero-variate data. See section on loss functions for the definition of the other symbols. MRE can have values in the interval [0, ∞], while SMSE has values in the interval [0, 1]. In both cases, 0 means predictions match data exactly. MRE assesses the differences between data and predictions additively, judging equally an overestimation and underestimation of the same relative size (e.g, +20% or −20% have the same contribution), while SMSE assesses the difference multiplicatively, judging overestimation and underestimation by the same factor equally (e.g. × 2 or ×1/2 have the same contribution). Notice that the result of the minimization of loss functions does not, generally, correspond with the minimum of MRE or SMSE (unless the fit is perfect).

### AmPtool

AmPtool is a software package that is designed to analyse patterns in (functions of) parameter values in selected entries. It is available via github.com/add-my-pet/AmPtool/, and changes frequently since the AmP collection is rapidly expanding. Meta-data, parameters and implied properties (biologically relevant quantities which are functions of parameters as well as food and temperature) are collected in a single Matlab structure (allStat.mat). Advanced plot-routines were developed to plot (functions of) parameters against other (functions of) parameters, for selected species. These selections make use of the taxonomic tree. The tree actually consists of lists-of-lists, based on the newest insights in taxonomy, as presented in the Catalog of Life, the Encyclopedia of Life and Wikipedia. Legends can be created where different taxomonic groups in the tree are attributed user-defined markers. This legend functionality allows selecting taxa at a large number of taxonomic levels. We mentioned in the previous section that goodness of fit is not enough to judge a set of DEB parameters and that one must also check for biological realism. AmPtool is one way to do this. The user can visualize how parameters of closely and less closely related taxonomic groups relate to each other and see if the new parameter estimates are extreme outliers or not. Careful examination of the coherence of the implied properties within an entry is the other way. AmPtool can also be used to find entries on the basis of data types that have been used, or print values of parameters and statistics of selected species.

## Results

### AmP web-interface

The AmP web-interface was developed as a result of this work. The interface allows examining each entry as well as obtaining on overview on how entries relate to each other.

#### A web interface for each entry

Each entry has its own web-interface that can be accessed via several routes, each organized differently (by classification in the species-list, according to phylogeny in the species-tree, alphabetical order of latin and english common names, and by energy budget, which is useful to walk from one species to a related one). The following information is printed for each entry in the online AmP web-interface: (i) (meta)data with references, (ii) predictions, (iii) (meta)parameters and (iv) implied properties. Meta-data (as introduced in the Methods) contains information on classification, authors, dates (submission, modification, acceptance), level of completeness of data, types of zero- and uni-variate data, discussion points about the fit (such as assumed differences between males and females) and facts about the species. Predictions of zero-variate data are presented as numbers, uni-variate data as graphs. Information on goodness of fit and model type is printed to the entry’s web-page. A separate page lists relevant implied properties, a list of (over 100) functions of parameters, representing physiological quantities (such as respiration) that might, or might not depend on food. The current and previous versions of the four user defined files (mydata, predict, pars_init and run) can be downloaded as .zip. Each entry is linked to relevant web-resources, e.g fishbase for fish species, amphiaweb for amphibians etc. A bib file with all of the bibtex references can also be downloaded. The acknowledgments and author names are also printed.

#### Web interface to compare species on the basis of their underlying metabolic properties

The **About** page presents a number of informative statistics of the AmP collection. The **Parameters** page gives an overview of the frequency distributions of 14 parameters that are shared by all models (Table 1) in the form of survivor functions, where the median values and the role of each parameter in the energetics of individuals is indicated. Parameters that depend on maximum structural length are scaled in a simple way such that they become independent of this length, since the collection covers the full range of 16 orders of magnitude of adult body weights among animals: from 4.10^−9^ g for gastrotrichs to 5.10^7^ g, for blue whales. This information can be used to judge parameter values of new species. The **Patterns** page illustrates some patterns in parameter values, such as (predicted) respiration as function of (predicted) maximum body weight. Four **Energy Budget** pages illustrate for all entries how allocation to somatic and maturity maintenance, to maturation (or reproduction) and to growth change during ontogeny.

### Comparing species on the basis of parameters

**Fig 3:**
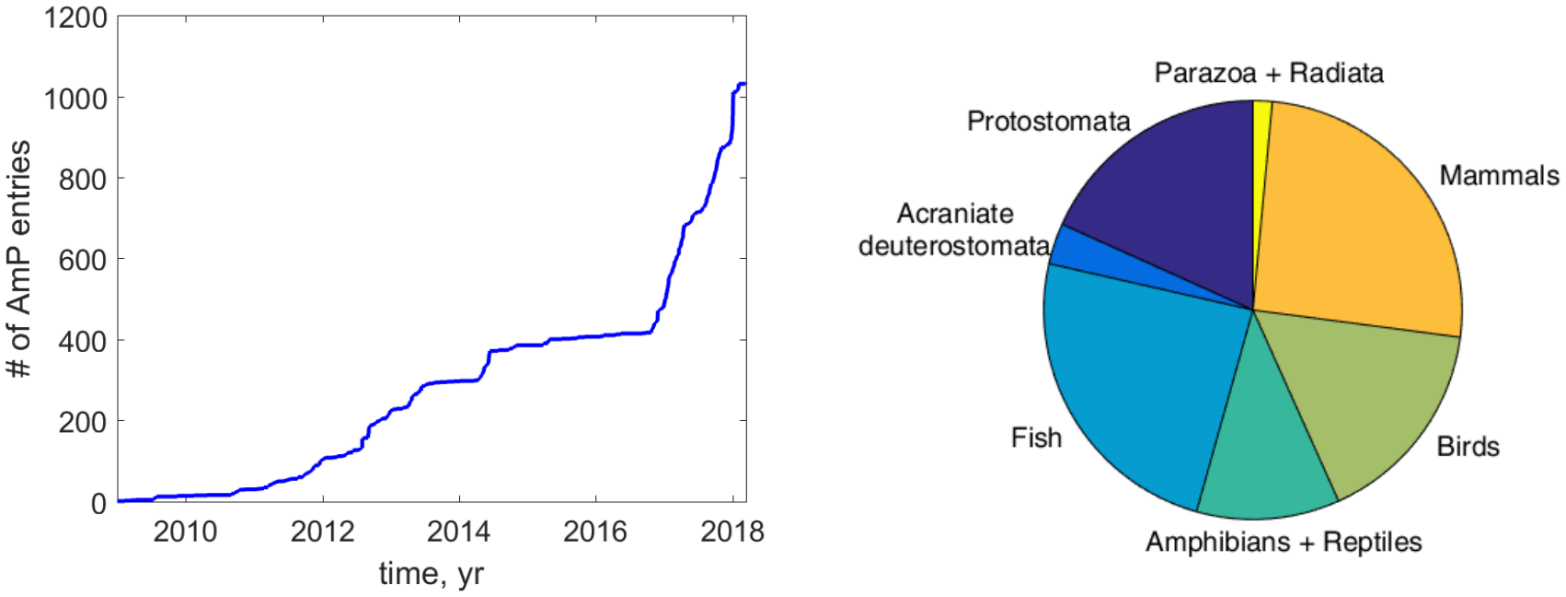
The number of species added to AmP in time and the relative frequency of taxa.

From 2013 till 2017 the number of AmP entries (Fig 3, left) was dramatically increased from ca 300 in 2013 to 1035 at 2018/03/12 to find out whether DEB models do apply to all animals and determine how problematic it is to have limited amounts of data for estimating DEB parameters. AmP receives entries which are submitted by the international scientific community and to date some 125 authors have contributed to the collection. Every author and their associated entry (with links to the entries) are listed on the AmP web-interface. Over the course of this study, we included a bit over 700 extra species to the collection since 2013 as well as converted the previous 300 entries to this new format. We were careful to select species such that the collection had a broad taxonomic scope, as well as included species of commercial relevance and species relevant for conservation and toxicity testing. We aimed at maintaining a good balance between the different taxonomic groups (Fig 3, right) We were on the look-out for exceptional species (in terms of size or aging) that might show that the DEB model was not applicable. We did not yet find such an animal species.

#### Improved estimation procedure

The new procedure allows selecting between several loss functions (see Methods). The “re” loss function, which was originally used, proved to be the cause of problems that we encountered with estimation: even when the estimation was started close to the global minimum, the estimate would sometimes move into the wrong direction. It is no longer recommended to use that option. We presented it here to give some historical context. The suitability of both “sb” and “su” were studied in technical companion study [32]. We adopted the “sb” as the default loss function for the AmP procedure and all of the parameters in AmP today use this loss function. Pseudo-data replace lack of information, serving as prior information on parameter values [22, 23]. The more information the data contain the less the pseudo-data contribute to the resulting parameter estimates. How can we check that the resulting parameters in AmP are not dominated by the use of pseudo-data? We studied how the DEB parameter “allocation fraction to soma”, *κ* (Table 1), was distributed across all AMP species (the graph is available on the parameters page of the AmP website). We refer the reader to [33] for an in-depth study of how *κ*, $\left[{\dot{p}}_{M}\right]$, $\left\{{\dot{p}}_{Am}\right\}$ and $\dot{v}$ are distributed across all of the species in AmP. Of relevance here, is that the values for the allocation fraction to soma, *κ*, in the collection turns out to follow a beta distribution with perplexing accuracy. The median value is 0.9, but the pseudo-data value for *κ* is 0.8. The explanation for why 0.8 is not the median value is that almost all entries have data on growth and reproduction, so *κ* is well determined by data, and the pseudo-data value then hardly plays a role. As mentioned in the methods, the default weight-coefficients for pseudo-data are set smaller than that for data to make sure that if data determine parameters well, pseudo-data hardly contribute. The default weight coefficients for the pseudo-data that are implemented in this new procedure are listed in Table 2 (these values can also be overwritten by the user). The motivation to decrease the weight coefficients of pseudo-data for “su” with respect to “sb” are detailed in [32]. With the introduction of the new features in the estimation method, and especially the use of filters, the method is now more robust, allowing to start further away from the final estimates. Several continuations (see Fig 2), using a maximum of say 500 steps, converges much more rapidly than a single run with a very large maximum step number. The reason is that the simplex typically shrinks rather fast, and a re-start (by continuation) restores the size of the simplex. This also reduces the risk of arriving to a local minima, rather than the global minimum of the loss function. The automatic computation of parameter values using the zero variate data for the species which is based on the bijection of nine data points to nine parameters [31] works in most cases, but not all. This functionality needs further testing, especially for models that differ from the standard DEB model. The feature can be useful for getting initial estimates.

#### Goodness of fit and completeness

**Fig 4:**
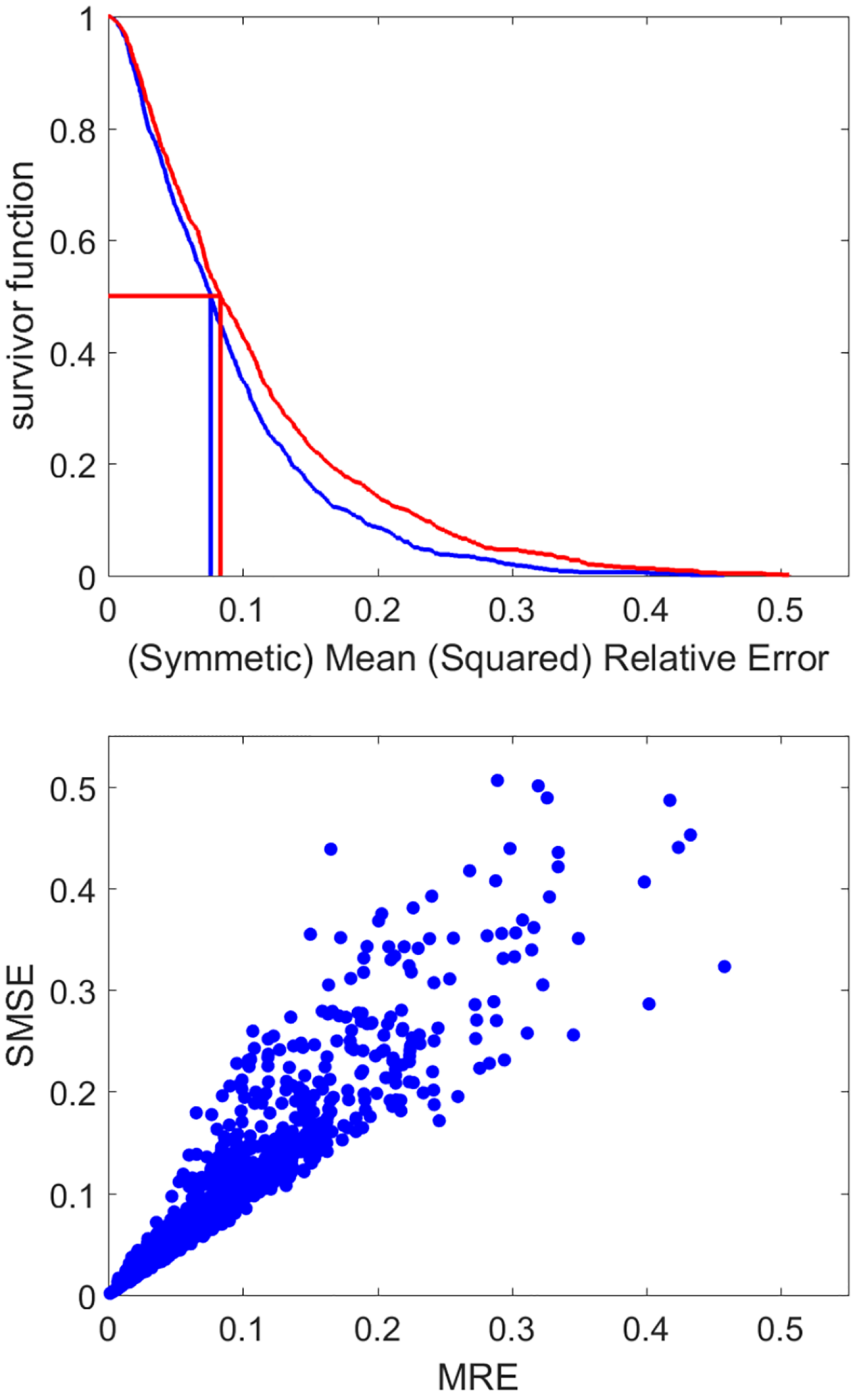
Goodness of fit of the DEB model to empirical data in the AmP collection: The survival function of MRE (blue) and SMSE (red), with their median values and the relationship between MRE and SMSE. See Methods for the definition of the MRE and SMSE.

**Fig 5:**
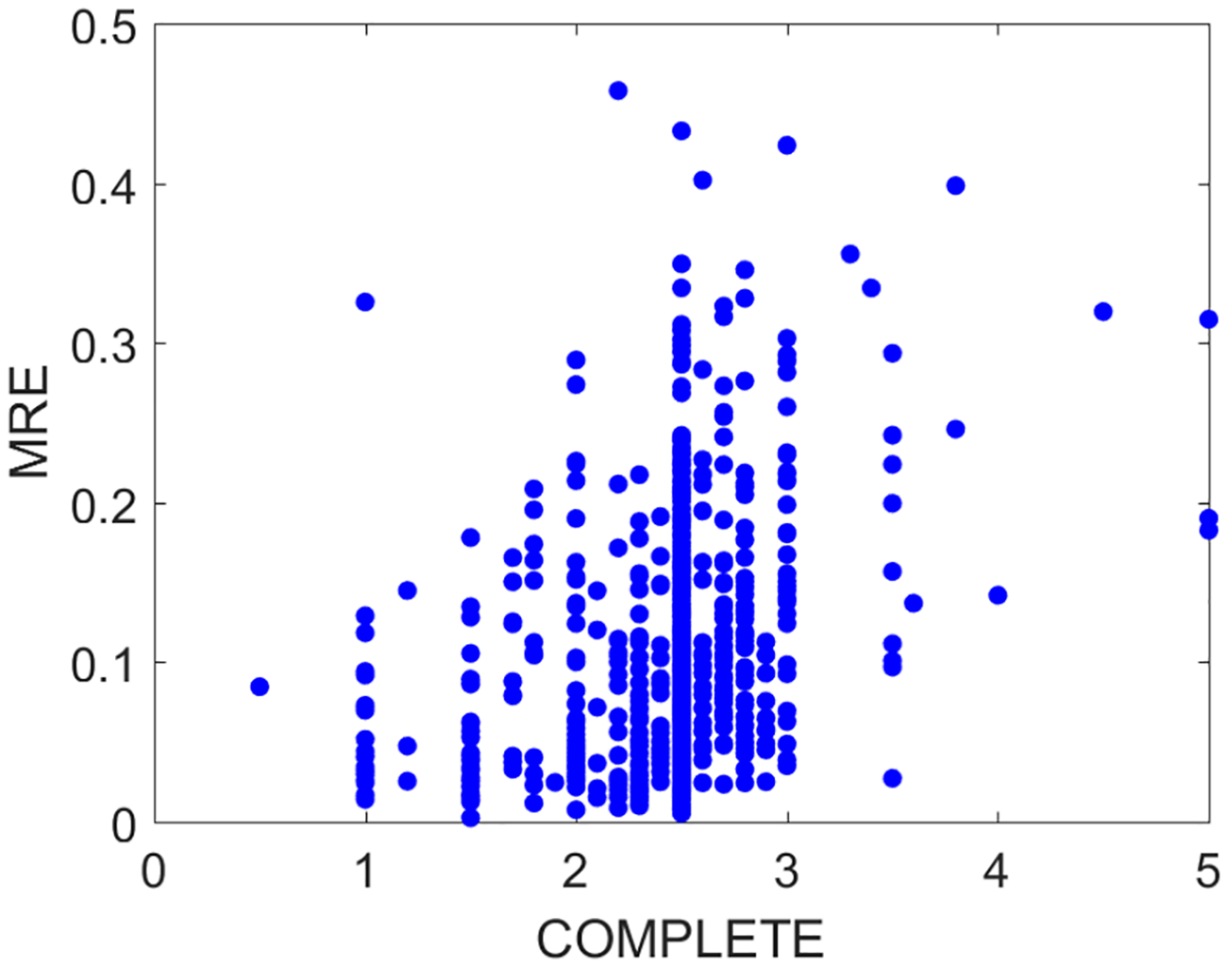
Model performance (in terms of mean relative error, MRE) with respect to the level of completeness of the data all parameters were estimated with loss function ‘sb’.

The Greenland shark [34] represents an example were very little data was available, but still allowed the estimation of parameters. While goodness of fit is not the only criterion for judging the parameters, it remains nonetheless important. For many applications it is very important that the model accurately predicts growth at different food and temperatures. The median MRE and SSME of predictions for all data for all species is less than 0.1 (see Fig 4). Data availability differs considerably among species (see Fig 5). Several entries concern extinct species, such as ten dinosaurs, a pterosaur, a giant crocodile, Archeopteryx and the great auk, illustrating that data does not need to be extensive to apply DEB models using our improved methodology. There is a tendency for the MRE to increase as function of the completeness of the data (Fig 5). Expanding on [35], deviations from predictions originate in decreasing order of importance: (i) lack of information on temperature and/or food availability/quality and how they change over time, which is replaced by simple assumptions, (ii) lack of details on how measurements have been done in detail, which is replaced by simple assumptions. For fish, for instance, standard, fork as well as total lengths are traditionally measured. However, what length measure was used is not always well indicated in the study, and each length measure needs to be linked to quantifiers for mass to allow application of DEB models. (iii) Different data belong to populations that differ in (mean) parameter values (e.g. because they experience different environmental conditions), but data availability does not allow parameter estimation for each population, (iv) intrinsic stochasticity of physiological/ecological behavior, partly originating from differences in parameter values between individuals. A large scatter translates to uncertainty in parameter values, but little scatter does not imply that parameter values are well determined due to model plasticity, see [32]. This aspect involves a new set of statistical problems, which are discussed in [32].

### Typified models

The increasing number of DEB applications on animal species motivated the continuing amelioration of the method, making it more robust, more efficient and easier to apply. As the collection of species grew, it became evident that the standard DEB model (Fig 1) required simple extensions for particular taxa, to accommodate larval life stages, fetal development, various forms of metabolic acceleration [36], substantial programmed shrinking (as sported by Elopocephalai), etc. DEB models are classified as s-models, a-models and h-models according to the mode of metabolic acceleration. Since we see the structured model collection that resulted from the AmP project as an important insight into animal metabolism, we come back to it in the discussion section. We here present the typified models. They must be understood as variations on the standard DEB model (std), see Methods section on DEB theory. The general idea is that the choice of typified model depends more on higher-level classifications than the species-level. Delayed stage transitions are also accounted for in the different model families. Most mammals delay the start of fetal development during gestation. Some bivalves delay the start of metabolic acceleration; this phenomenon can prove to be more common with the increase of available data. The three sets of models are detailed below.

#### s–models

The **s**–models apply to most animal species without larval phases. Models for mammals are part of this model family but deviate from the standard model by having a fetus, the production of milk mostly by females and a diet-switch of the juvenile at weaning. Most mammals also delay start of fetal development during gestation. The s–models have been classified as follows:

- std: standard DEB model with egg development
- stf: std but with fetal development (rather than egg development).
- stx: Like model stf but with fetal development that first starts with a preparation stage and then sparks off at a time that is an extra parameter (*t*_0_, d). The stf includes a baby stage (for mammals) just after birth, ended by weaning, where juvenile switches from feeding on milk to solid food at maturity level $E_{H}^{x}$. Weaning is between birth and puberty. In its simplest form, it is a two parameter extension of model std at abundant food. Food quality and up-regulation can involve more parameters.
- ssj: Like model std but with a non-feeding stage between events s and j during the juvenile stage that is initiated at a particular maturity level and lasts a particular time. Substantial metabolically controlled shrinking occur during this period, more than can be explained by starvation. It is a two or three parameter extension of model std. This life history is found in Elopiformes, Albuliformes, Notacanthiformes, Anguilliformes, Ophidiiformes, some Anura and Echinodermata.
- sbp: Like model std but growth ceasing at puberty, meaning that the kappa-rule is not operational in adults. It has the same parameters as the model std. This life history is found in the one Calanus entry, while other copepods in AmP accelerate (see next section). We obviously need more data and better culturing techniques to become better organised on copepod models.

#### a–models

The **a**–models apply to most species with a larval phase. They show metabolic acceleration at, or soon after, birth; the end of acceleration frequently coincides with morphological metamorphosis. s–models assume isomorphic growth over all life stages. DEB theory assumes that assimilation increases with surface area and maintenance with volume, but makes no assumptions on how surface areas relate to volume. Isomorphy entails constant surface-area to volume ratio and so implies that growth slows down because the incoming energy can no longer come in faster that it is burned for maintenance. Organisms have leeway to play with how structural surface area relates to volume by modifying their structural shape during ontogeny. If they increase their surface proportional to volume (the technical term is V1-morphy) then this impacts both how fast it assimilates food as well as how fast it mobilizes energy (with respect to an isomorph). Assimilation continues to increase with volume. Mobilization increases because reserve mobilization is proportional to the surface area of the reserve to structure interface.

All a-models assume that metabolism accelerates during part of the life-cycle following the rules for V1-morphy.

- abj: is like model std, except that metabolic acceleration occurs between birth and metamorphosis. Before and after acceleration growth is isomorphic. Metamorphosis is before puberty and occurs at maturity level $E_{H}^{j}$ (J), which might or might not correspond with changes in morphology. This model is a one-parameter extension of model std.
- asj: is like model abj, but the start of metabolic acceleration is delayed till maturity level $E_{H}^{s}$. Metamorphosis is still before puberty. This model is a one-parameter extension of model abj.
- abp: is like model abj, except that metabolic acceleration occurs between birth and puberty and that after acceleration there is no growth, so no kappa-rule. A morphological metamorphosis can occur before puberty at maturity $E_{H}^{j}$, but this only affects morphology, not metabolism. This model has the same number of parameters as model std. It applies to copepods, and possibly applies to ostracods, spiders and scorpions.

#### h–models

h-models are as a-models, but with extra life stages (as found in insects) triggered by the reproduction buffer density, i.e. the amount of reproduction buffer per unit of structure (see Methods, DEB theory). The **h**–models mostly apply to insects (hexapods).

- hep: DEB model for ephemeropterans, odonata and possibly other insect groups. It specifies the following morphological life stages: egg, larva, (sub)imago; functional stages: embryo, juvenile, adult, imago. The embryo still behaves like model std, acceleration starts at birth and ends at puberty. Puberty (i.e. the onset of energy allocation to a reproduction buffer) occurs during the larval stage. Emergence occurs when reproduction buffer density hits a threshold, the (sub)imago does not grow or allocate to reproduction. It mobilizes reserve to match constant (somatic plus maturity) maintenance.
- hex: this is the DEB model for holometabolic insects (and some other hexapods). It specifies the following morphological life stages: egg, larva, (pupa), imago; functional stages: embryo, adult, (pupa), imago. The embryo still behaves like model std while the larval stage accelerates (V1-morph) and behaves as adult, i.e. no maturation, but allocation to reproduction. Pupation occurs when the reproduction buffer density hits a threshold. The pupa behaves like the embryo of std, except that larval structure rapidly transforms to pupal reserve just after the start of pupation. The reproduction buffer remains unchanged during the pupal stage. The imago does not grow or allocate to reproduction. It mobilizes reserve to match constant (somatic plus maturity) maintenance.
  Hemi-metabolic insects skip the pupal stage, do not convert larval structure to reserve. Imago structure equals larval structure when reproduction buffer density hits a threshold.

#### Overview of typified models in AmP

**Fig 6:**
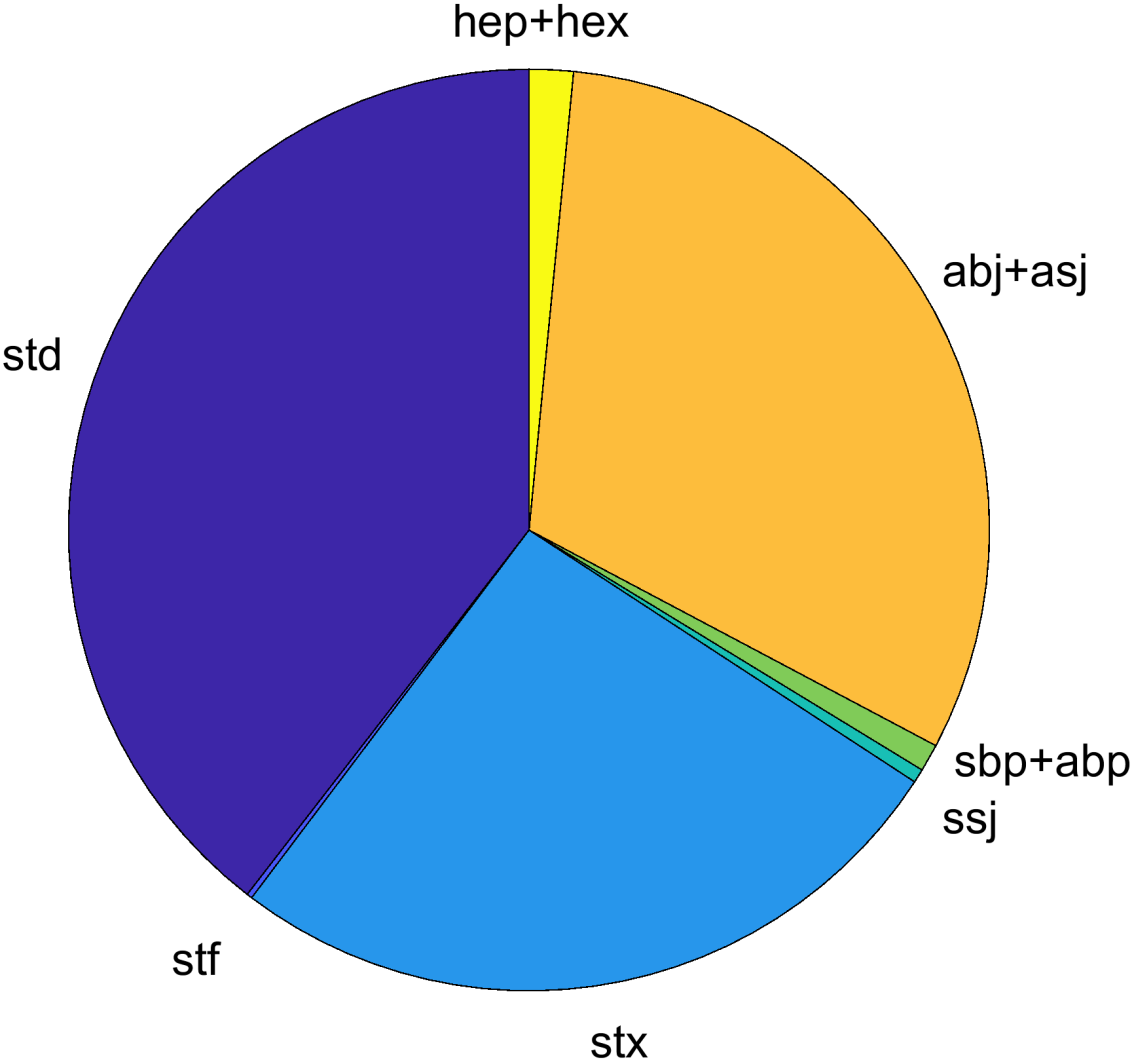
Statistics of the AmP collection: The relative frequency of standard-like (s-), acceleration (a-), and insect-like (h-) models.

The pie in Fig 6 presents their relative frequency in the collection. Certain typified models are better resolved that others, because of number of entries and data with the target groups.

## Discussion

### Comparisons of species on the basis of parameter values

The AmP database not only allows us to test model against data and evaluate implications, but also to identify evolutionary and ecological patterns in parameter values. The number of papers on patterns in parameter values is now increasing rapidly [10, 15, 30, 31, 33, 36–41]. Apart from being of direct scientific relevance, future improvements of DEB parameter estimation methods might exploit these patterns, since lack of data is the rule rather than the exception and having some testable prediction is better than no prediction at all.

Where, for instance, (measured) maximum body weight is treated as an independent variable in the eco-physiological literature, DEB theory sees this as a property resulting from underlying processes that are quantified via parameter values. So maximum body weight is a function of parameter values (and food availability). Respiration (e.g. the use of dioxygen) is another function of parameter values, which has contributions from various underlying processes, such as maintenance, development, growth and assimilation overheads, etc. While respiration has been measured for only a small part of the species in the AmP collection, it is available for all species as a prediction (i.e. a function of parameter values). This is just an illustration of the power of comparison on the basis of parameter values, rather than on the basis of measurements. The **Patterns** page on the AmP website illustrates some patterns in parameter values, such as (predicted) respiration as function of (predicted) maximum body weight, confirming Kleiber’s empirical law stating that (measured) respiration is about proportional to (measured) maximum weight to the power 3/4. Two other patterns illustrate the explanation provided by DEB theory: relative reserve capacity is increasing and specific somatic maintenance is decreasing with body size. Reserve does not require maintenance, so does not contribute to respiration. The increase of reserve capacity $\left[E_{m}\right]=\left\{{\dot{p}}_{Am}\right\}/\dot{v}$ with maximum (structural) length follows from co-variation rules [29], where energy conductance $\dot{v}$ (which quantifies reserve mobilization) is independent of maximum length, and specific maximum assimilation rate $\left\{{\dot{p}}_{Am}\right\}$ is proportional to it. The increase of specific somatic maintenance with decreasing maximum (structural) length is seen as an ecological adaptation to exploit short-lasting peaks in food abundance [36]. Another pattern shows that, contrary to popular believe, the maximum growth rate of dinosaurs is just in line with other taxa, given their body size. The full analysis of all patterns in parameter values is beyond the scope of this work, and is an ongoing activity [33, 41].

### Model types and evolution

The classification of DEB models into s–, a– and h–models has more or less clear links with the evolution of metabolism. This will be discussed here using a feature that sets a– and h–models apart from s–models: metabolic type M acceleration [36], where surface area temporarily scales with volume, while before and after this period it scales with volume to the power 2/3. The amount of type M acceleration is quantified by the ratio of body length at the end and the start of the acceleration period. The effect of this change in a DEB context is that both the specific assimilation and the energy conductance increase with length, while outside the acceleration period they stay the same. The ratio of the two, the specific reserve capacity, is not affected by this type of acceleration. The h–models differ mainly from the a–models by the fact that acceleration extends into the adult stage.

**Fig 7:**
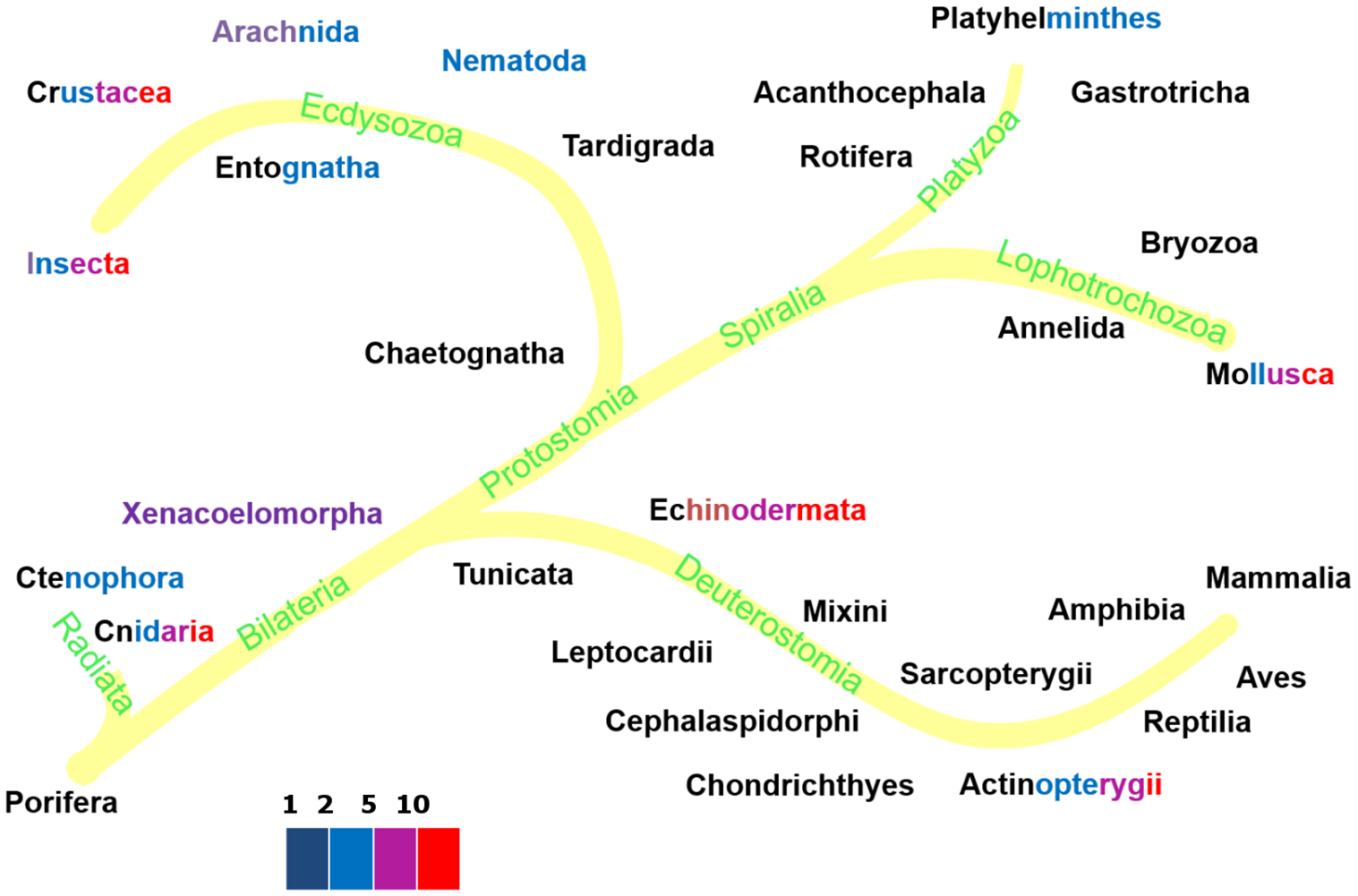
The metabolic acceleration factor in animal taxa, quantified as the ratio of length after and before acceleration. The font colors of the taxa names indicate the values of the acceleration factor among their species: less than 2 (black), 5 (blue), 10 (magenta) or more than 10 (red).

Fig 7 presents evolutionary relationships among animal taxa with a color code to indicate the amount of acceleration in their species. Since the oldest animal group, the Radiata, and the oldest deuterostomes, the echinoderms, accelerate, it might well be that acceleration became suppressed in several other groups and this suppression evolved several times in evolution [36].

**Fig 8:**
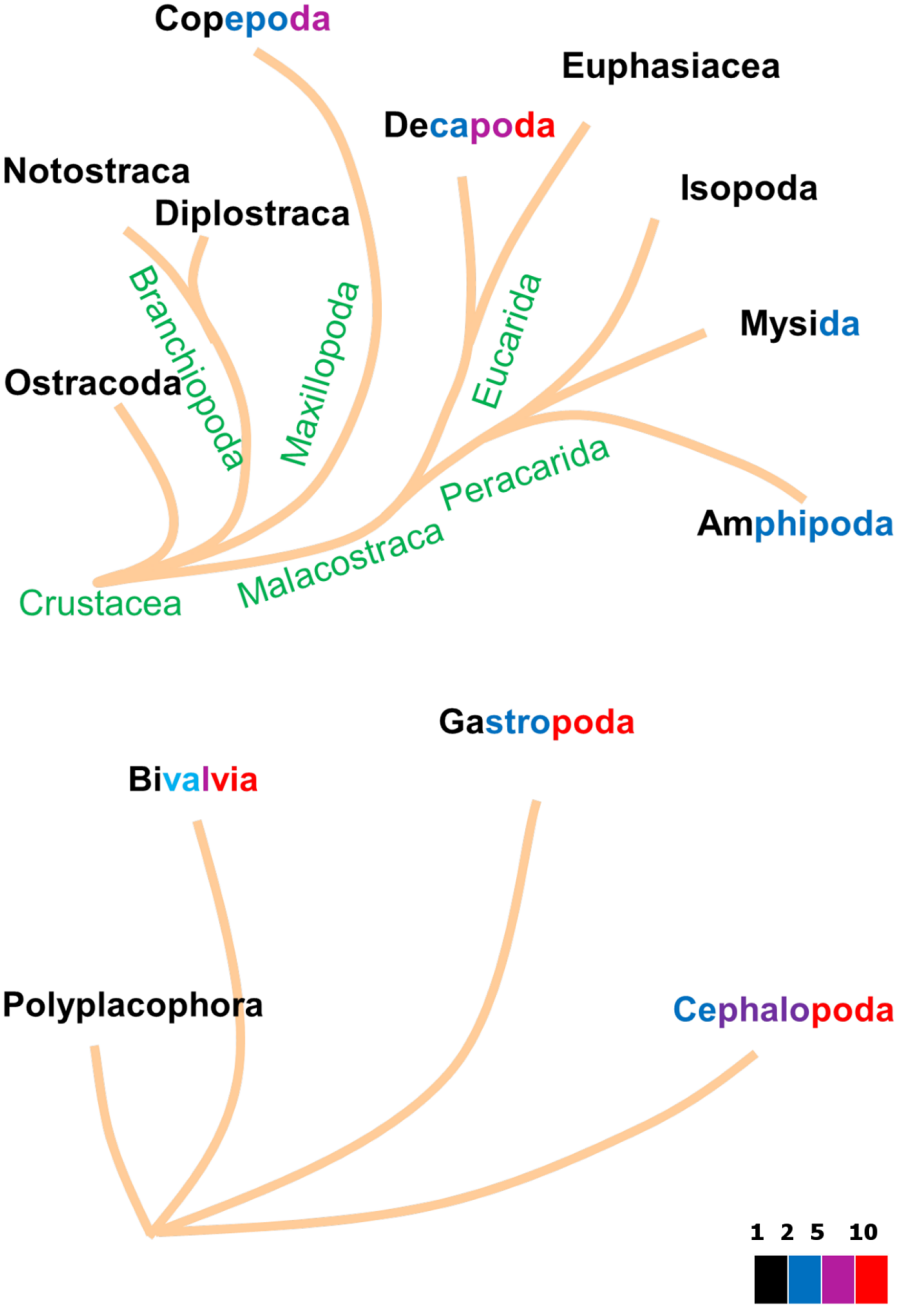
The acceleration factor, i.e. the ratio of lengths after and before acceleration, for the various taxa of Crustacea (top) and Mollusca (bottom). The font colors indicate acceleration by a factor less than 2 (black), 5 (blue), 10 (magenta) or more than 10 (red).

**Fig 9:**
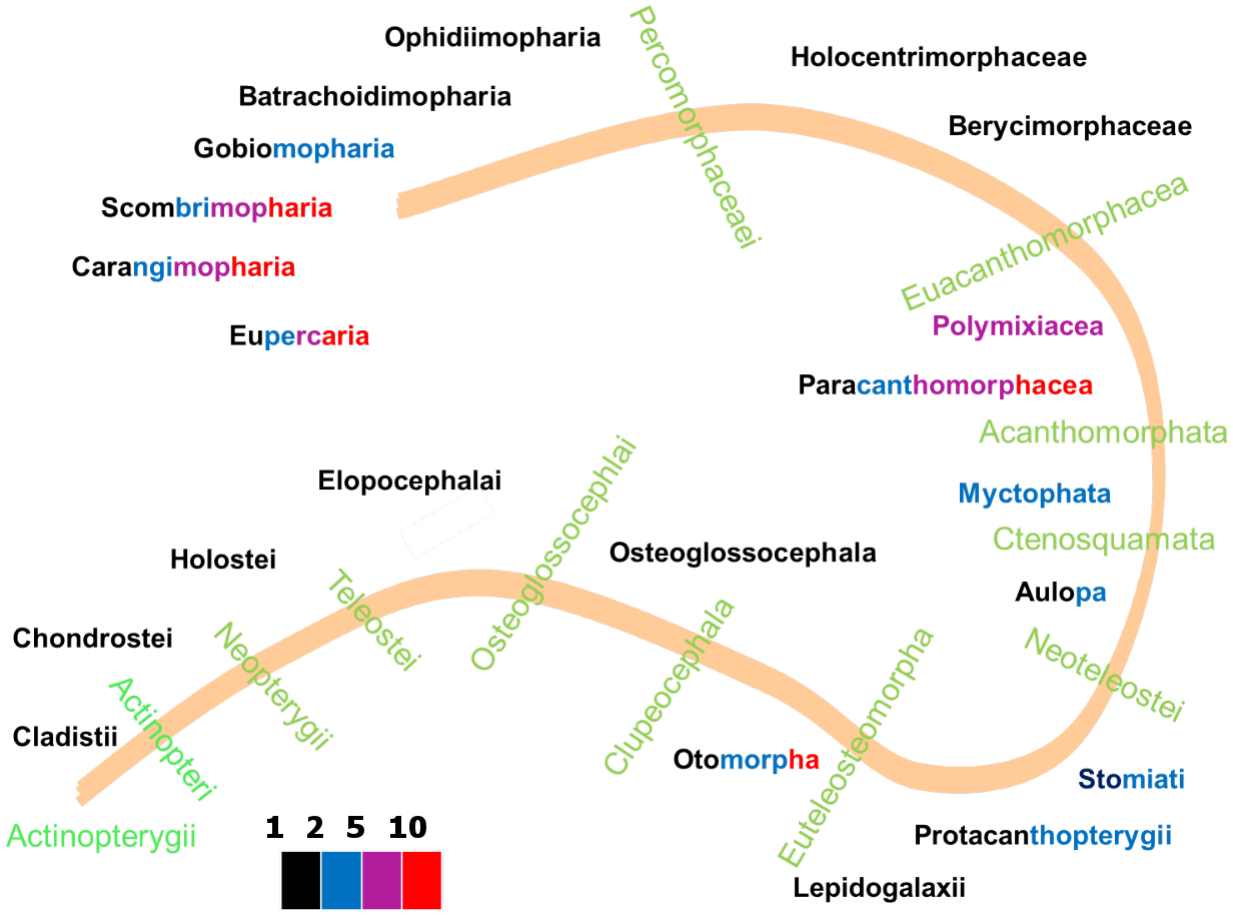
The acceleration factor, i.e. the ratio of lengths after and before acceleration, for the various taxa of the ray-finned fish. The font colors indicate acceleration by a factor less than 2 (black), 5 (blue), 10 (magenta) or more than 10 (red).

The Ecdysozoa (*n* = 99 at 2018/01/01) beautifully illustrate the link between model type and taxonomic relationship. Fig 7 shows that Chaetognatha, Tardigrada, Nematoda and Entognatha hardly accelerate metabolism, just a factor 2 or less. The basic insects, the ephemeropterans and odonata accelerate a bit more, while the crown groups, the holometabolic insects, accelerate very much. All of the h–models are found in insecta, but very interestingly, springtails Entognatha (*n* = 6), which are no longer classified as insects, follow the abj model. Most insects seem to skip the juvenile phase and allocate to reproduction as larvae, which classifies them as adult by definition in DEB terms, while the imago neither grows, nor eats (frequently). Holometabolic insects insert a pupal phase between the larval and imago phases that behaves like an embryo with a reproduction buffer, where most of the larval structure is first converted to reserve [42] and imago structure is build from reserve. Crustacea (*n* = 62) sport a mix of s– and a–models Branchiopoda (*n* = 18) are described by std. Copepoda (*n* = 6) are hardly resolved and require more research. Calanaus does not accelerate (sbp), the others do (abp). Copepods are special with respect to the other crustaceans in that the *κ*-rule no longer applies to the adult stage. The 1 species of ostracod is described by abj. Malacostraca are better represented in the collection (*n* = 36) and are described by abj. While abp now applies to most copepods, it may be that it also applies to ostracods, arachnids and scorpions. The future will teach us. The results further show that among the spiralia only some mollusk species accelerate metabolically, see Fig 8. Ray-finned fish (*n* = 206) sport a wide range of acceleration factors, see Fig 9, but extreme forms of acceleration are confined to the Otomorpha, the Paracanthomorphacea and the crown groups of the Percomorphaceaei only. The coupling between the amount of acceleration in mediterranean perches with the spawning season, as reported in [39], shows that, apart from evolutionary aspects, ecological ones are involved as well and the two aspects cannot be fully separated. These examples beautifully show that the occurrence of acceleration is far from random.

Energy conductance, one of the two parameters that are affected by metabolic acceleration, controls reserve mobilisation, so dominates the incubation (or gestation time), since eggs start their development as a lump of reserve. We have several cases with data for embryo development in combination with post natal development, which show that energy conductance remains constant before and after birth. We met one convincing case, the Asian freshwater leech, *Barbronia weberi* [43], where energy conductance makes a jump up at birth. There are quite a few cases, however, where incubation time is under-estimated. These cases do not have data on embryo development, so we cannot be sure if energy conductance also makes a jump here, or that the start embryo development is delayed. The latter might be due to a variety of reasons. Most mammalian embryos, for instance, have a period to prepare for growth during which the fetus does not increase in size. The onset of growth is typically quite clear, since consistent with DEB expectations, structural length starts to increase linearly [44, 45]. Data on embryo development is relatively scarce.

The colors in the taxa names of Figs 7, 8 and 9 reflect the range of values of metabolic acceleration that were found in the various taxa. Although over 1000 entries is very large for a database of this type, compared to the 10 million existing animal species, it is close to nothing. Thus we cannot assume that the species in the collection are fully representative for the species in nature. Moreover, the number of species in each taxon, both in nature and in the database, varies enormously; some have just a single species. It is already an accomplishment to indicate the ranges this way, and we will not be surprised if insights on which taxa accelerate change somewhat as the collection grows. Since the database is online and freely accessible, the reader can easily check the values of metabolic acceleration for each species in each taxonomic group using the free package AmPtool (we refer the reader to the online manual for how to do this).

### Conclusions

This study represents a large scale application of a general theory for metabolic organization of living organisms: the Dynamic Energy Budget theory. Although DEB theory applies to all organisms, the AmP collection only deals with animals. The reason is that animals eat other organisms, which do not vary much in chemical composition. As a first approximation, their environment can be characterized by the variables food availability and temperature. This characterization is hard to make “complete” for other organisms, which hampers comparison. And comparison is the most useful asset of this collection.

We contend that animal species can be compared on the basis of DEB parameters and that this offers a tractable means to study animal biodiversity in an ecological and evolutionary context. Moreover, by being mechanistic (= based on first principles), DEB models interpret data, rather than just describe it. They can therfore reveal inconsistencies in data and predict un-measured properties of species as functions of parameters.

We present and discuss how DEB parameters can be extracted from eco-physiological data: the AmP approach. The two associated software packages, DEBtool and AmPtool, are freely available via GitHub and have online user manuals. We demonstrate the applicability of DEB theory, by showing that it is possible to extract DEB parameters for animal species even when there is little data. We evaluate goodness of fit with respect to data completeness per species and overall the models fit data well. We found that a family of related DEB models, which share the same 14 DEB parameters, are needed to capture the diversity of life-cycles in the animal kingdom. A main metabolic feature which distinguishes the life-cycles is that some groups have ‘metabolic acceleration’, which has links with larval stages. We present the latest evolutionary overview on which groups were found to have metabolic acceleration. Knowledge gaps are highlighted.

The AmP project was initiated at 2009/02/12 and meanwhile 125 authors contributed with entries. It has 1035 entries at 2018/03/12, including all larger phyla and all chordate orders, and is both the smallest as well as the largest database of this kind, since it is unique. We expect that it will remain unique for a long time to come, in view of the huge amount of effort to arrive at the state we are presently in and because we think that DEB models will not have alternatives with matching generality, simplicity and realism. We hope that this study motivates the scientific community to contribute and use the AmP collection.
